# Critical Windows of Prenatal Exposure to Cadmium and Size at Birth

**DOI:** 10.3390/ijerph14010058

**Published:** 2017-01-09

**Authors:** Lu Cheng, Bin Zhang, Tongzhang Zheng, Jie Hu, Aifen Zhou, Bryan A. Bassig, Wei Xia, David A. Savitz, Stephen Buka, Chao Xiong, Joseph M. Braun, Yaqi Zhang, Yanqiu Zhou, Xinyun Pan, Chuansha Wu, Youjie Wang, Zhengmin Qian, Aimin Yang, Megan E. Romano, Kunchong Shi, Shunqing Xu, Yuanyuan Li

**Affiliations:** 1 Key Laboratory of Environment and Health (HUST), Ministry of Education and Ministry of Environmental Protection, School of Public Health, Tongji Medical College, Huazhong University of Science and Technology, Wuhan 430000, Hubei, China; chenglu822@hotmail.com (L.C.); mchwhzb@163.com (B.Z.); hujie_tjmu@126.com (J.H.); xiawei@hust.edu.cn (W.X.); 16483596@life.hkbu.edu.hk (Y.Z.); panxinyun721@163.com (X.P.); shalovesafin@126.com (C.W.); wangyoujie@mails.tjmu.edu.cn (Y.W.); xust@hust.edu.cn (S.X.); 2 State Key Laboratory of Environmental Health (Incubation), School of Public Health, Tongji Medical College, Huazhong University of Science and Technology, Wuhan 430000, Hubei, China; 3 Women and Children Medical and Healthcare Center of Wuhan, Wuhan 430000, Hubei, China; april1972@163.com (A.Z.); xcpopo11@yeah.net (C.X.); cardinal3542@hotmail.com (Y.Z.); 4 Department of Epidemiology, Brown University, Providence, RI 02912, USA; tongzhang_zheng@brown.edu (T.Z.); david_savitz@brown.edu (D.A.S.); stephen_buka@brown.edu (S.B.); joseph_braun_1@brown.edu (J.M.B.); megan_romano@brown.edu (M.E.R.); kunchong_shi@brown.edu (K.S.); 5 Department of Environmental Health Sciences, Yale School of Public Health, New Haven, CT 06520, USA; bryan.bassig@nih.gov; 6 Department of Epidemiology, College for Public Health and Social Justice, Saint Louis University, Saint Louis, MO 63103, USA; zqian2@slu.edu; 7 College of Earth and Environmental Science, Lanzhou University, Lanzhou 730000, Gansu, China; aimin_yang@brown.edu

**Keywords:** cadmium, birth weight, fetal exposure, critical window, epidemiology

## Abstract

Prenatal cadmium (Cd) exposure has been associated with adverse birth outcomes, but the findings of previous studies are inconsistent. We measured Cd concentrations in urine samples at or near 13, 24, and 35 gestational weeks from 282 women in Wuhan, China. We used generalized estimating equation models to assess the associations between maternal creatinine adjusted urinary Cd concentrations at each trimester and birth size. A significant inverse association was observed between higher maternal Cd levels measured during the 1st trimester and birth size in girls. For each log unit increase in Cd (µg/g creatinine) levels from the 1st trimester, there was a decrease in birth weight by 116.99 g (95% confidence interval (CI): −208.87, −25.11 g). The Cd levels from the 1st and 2nd trimesters were also borderline significantly associated with ponderal index in girls. Joint estimation of trimester-specific effects suggested that associations with Cd levels for ponderal index (*p*_int_ = 0.02) were significantly different across trimesters, and differences for effects across trimesters for birth weight were marginally significant (*p*_int_ = 0.08) in girls. No significant associations were observed between Cd levels from any trimester and birth size in boys. Maternal Cd exposure during earlier periods of pregnancy may have a larger impact on delayed fetal growth.

## 1. Introduction

Cadmium (Cd) is a toxic heavy metal that is ubiquitously distributed in the environment, mainly as a result of pollution from many modern industrial processes. Environmental exposure to Cd in humans mainly results from smoking, ambient air, and the ingestion of contaminated food (e.g., grains, vegetables, offal, and seafood) [1]. In adults, exposure to Cd has been known to adversely affect the kidneys, lungs, and bone, and has been linked to an increased risk of cancer and overall mortality [2,3,4]. The International Agency for Research on Cancer has concluded that there is sufficient evidence in humans that Cd and Cd compounds are carcinogenic, with particularly strong associations with lung cancer [5].

Cd is primarily stored in the liver and kidneys, however, the placenta also accumulates Cd [6], which may lead to decreased uteroplacental blood flow [7] or affect the synthesis and metabolism of placental hormones [6]. In addition, there is still a fraction of maternal Cd can reach the fetus from the pregnant woman through transplacental, which may disrupt the fetal growth. The accumulated Cd in the placenta and the transplacental Cd both may disrupt fetal development. This has led to increasing interest in evaluating the relationship between prenatal Cd exposure and birth outcomes. However, the majority of earlier epidemiologic studies have relied on Cd levels measured at delivery to assess the cross-sectional association between prenatal Cd exposure and birth outcomes, and have produced conflicting results. For example, some studies have reported that Cd levels in maternal blood collected at delivery [8] or cord blood [9] were associated with reduced weight or head circumference at birth, whereas other studies have not observed associations between size at birth and Cd levels that were measured in the placenta [10,11] or in maternal blood that was collected during the third trimester [12]. One study using one spot maternal urine sample collected in early pregnancy found a sex difference in the association between maternal Cd exposure and birth size, which was apparent only in girls [13]. However, a study from Canada found that the average of blood Cd concentrations from the first and third trimester was not associated with risk for small-for-gestational age (SGA) [14]. Thus, our knowledge of the effects of prenatal Cd exposure on birth outcomes remains limited and inconsistent.

The fetal period is considered to be a sensitive stage of human development to environmental exposures, and the vulnerability of the fetus to environmental exposures in different pregnancy stages may be considerably different [15]. Exposures occurring at different stages of pregnancy, even at comparable levels, may have very different impacts on fetal development and birth outcomes. This suggests the presence of critical windows of susceptibility to environmental exposures, like Cd, and highlights the importance of identifying these windows of vulnerability. The inconsistent findings of prior epidemiologic studies that have studied birth outcomes in relation to prenatal Cd exposure may be partially attributed to this variability in the assessment and timing of fetal Cd exposure.

In this cohort study, we assessed the trimester-specific effects and the sex difference of prenatal exposure to Cd in relation to size at birth using maternal urinary Cd levels measured at each pregnancy trimester in 282 singleton pregnant women from Wuhan, China.

## 2. Materials and Methods

### 2.1. Study Subjects

The present study was carried out between October 2013 and October 2014 at the Wuhan Women and Children Medical Care Center, a major maternity hospital in Wuhan, China. A total of 282 pregnant women who satisfied the following conditions were recruited: (1) aged 20 years or older; (2) coming to the hospital for their first prenatal examination; (3) less than 16 weeks pregnant with a singleton gestation at the time of enrollment; (4) residence in Wuhan City at the time of the recruitment period; and (5) willingness to have prenatal care and give birth in the study hospital. All participants in this study provided written informed consent at enrollment. The research protocol was approved by the ethics committee of the Tongji Medical College, Huazhong University of Science and Technology (No. (2012)07), and the Wuhan Women and Children Medical Care Center (No. 2012003).

### 2.2. Exposure Assessment

Maternal urine samples were collected during prenatal visits to the hospital at each trimester. On average (±SD), the 1st, 2nd, and 3rd trimester gestational ages at urine collection were 13 ± 1.2, 24 ± 2.9, and 35 ± 4.4 weeks. Among the 282 women who were enrolled in the study, all of the women donated at least two urine samples, and 238 (84%) donated one urine sample at each trimester. The number of women who did not donated urine samples during the first, second, and third trimester was 3, 36, and 6, respectively. The reason that pregnant women did not provide urine samples was they did not go to the hospital for prenatal care within the specified time. There was no difference between pregnant women who provided urine samples and those who did not.

Urine samples were collected in polyethylene containers and stored at −20 °C until further analysis. The sample preparation and analytical methods have been described elsewhere [16]. Briefly, prior to analysis, urine samples were thawed at room temperature, and 0.5 mL of urine was added to 15 mL Kirgen polypropylene conical centrifuge tubes. Then, 1.2% (v/v) HNO_3_ was added to the final volume of 2.5 mL for overnight nitrification. The resulting sample was digested at 40 °C for 1 h and then analyzed for Cd. Cd in urine was measured by inductively coupled plasma mass spectrometry (ICP-MS) (Agilent 7700, Agilent Technologies, Santa Clara, CA, USA) in helium mode. The operation conditions of ICP-MS were as follows: RF power 1550 w, plasma gas flow 15.00 L/min, auxiliary gas flow 0.9 L/min, carrier gas flow 0.25 L/min, resolution (peak high 10%) 0.65~0.80 atomic mass unit (amu), improve quantity of samples 0.4 mL/min, unimodal residence time 0.1 s.

Quality control urine samples were incorporated in each batch of 20 samples and each quality control sample was measured in duplicate. All available urine samples from each mother were measured in the same batch. National Institute of Standards and Technology (Gaithersburg, MD, USA) Standard reference material was used as an external control in each batch, and the concentrations measured were within the range recommended by the manufacture (5%). The limit of detection was 0.002 µg/L, and no samples were below it. The spiked recovery of the quality control standard was 103%. The between-assay coefficient of variation (CV) was 2.2% and the within-assay CV was 1.1%.

Urinary creatinine, determined by a creatinine kit (Mindray BS-200 CREA Kit, Mindray Bio-medical Electronics Co., Ltd., Shenzhen, China), was used to control for variations in urine dilution in spot urine specimens. The adjusted urinary Cd concentration was expressed as µg/g creatinine.

### 2.3. Outcomes and Covariates

The following measures of size at birth were obtained: birth weight, birth length, and ponderal index. Birth weight (in grams) and birth length (in centimeters) were retrieved from medical records. The measurements of birth weight and birth length in the hospital were conducted within one hour after birth by experienced obstetric nurses using standardized procedures. Ponderal index, an indicator of fetal growth status, especially to assess asymmetrical intrauterine growth retardation, was calculated using the following formula: (weight in grams/length^3^ in centimeters) × 100 [17].

Covariate data including demographic (date of birth, ethnicity, and marital status), socioeconomic (education, occupation, household income), and lifestyle factors (alcohol and tobacco exposure) were obtained through face-to-face interviews with each mother. Maternal smoking was defined as any smoking while pregnant based on self-report. Passive smoking was defined as exposure of a nonsmoker to tobacco smoke more than 30 min per day during pregnancy. Maternal drinking was defined as a frequency of drinking of more than one time per week during pregnancy. The interviews were conducted in the hospital by specially trained nurses within three days before or after delivery. Maternal weight before pregnancy and at delivery, maternal height, and information concerning the infants’ birth date, gender, and gestational age were all retrieved from medical records. Gestational age was calculated by subtracting the date of the last menstrual period (LMP) from the date of birth. For women who had a regular menstrual cycle and could report the accurate date of the LMP, the reported LMP was used when the difference between the reported gestational age and ultrasound-based gestational age was within 7 days. For women who had an irregular menstrual cycle and could not report the accurate date of the LMP, the ultrasound-based LMP estimated by experienced obstetricians was used.

### 2.4. Statistical Analyses

Descriptive statistics were performed prior to bivariate analyses. Urinary Cd levels in the three trimesters were compared using the Kruskal-Wallis test, and the correlations between Cd levels in different pregnancy stages were determined by Spearman correlation tests. The Wilcoxon rank sum test was used to compare distributions of Cd levels between mothers of boys and girls. Bivariate analyses among exposures, outcomes, and covariates were assessed using Spearman correlation coefficients.

We assessed the associations between urinary Cd concentrations and birth outcomes (birth weight, birth length, and ponderal index) using a generalized estimating equation (GEE) model with a linear link function [18]. The GEE approach embeds separate linear regression models for each trimester into a unified set of estimating equations, which allows for the assessment of whether the exposure coefficients were equal across the three trimesters by integrating the measurements of the three trimesters into the model. We used log-transformed creatinine-adjusted Cd concentrations to reduce the influence of outlying Cd values. Variables considered to be potential confounders were selected based on either biologic plausibility or those variables that were significantly (*p* < 0.1) associated with either urinary Cd or birth size in the bivariate analyses. Maternal education and household income were correlated (*p* < 0.001). Therefore, we adjusted for maternal education only because it was associated with birth size more strongly than household income. As most of the women have never given birth before (primiparous, 87.9%) and parity was not associated with urinary Cd or birth size, we did not adjust for parity. Based on these criteria, confounders that were included in the subsequent GEE models included newborns’ sex, gestational age, maternal age at delivery, maternal BMI before pregnancy, net weight gain during pregnancy (maternal pregnancy weight gain minus the newborn′s weight), maternal education level, and passive smoking. Maternal height was used instead of BMI before pregnancy for the birth length analysis, because it has been reported to be strongly associated with birth length [19].

A series of sensitivity analyses were conducted. We first repeated analyses with the exclusion of women whose gestational age was less than 37 weeks. Then, we assessed whether including calcium supplementation into the models modified the associations between Cd and birth size. In addition, we ran models that incorporated log-transformed creatinine concentrations as a covariate rather than using creatinine-adjusted Cd concentrations. All the statistical analyses were performed with SAS (version 9.4; SAS Institute Inc., Carry, NC, USA). A *p*-value < 0.05 was considered statistically significant.

## 3. Results

The mean age of the women at delivery was 28.8 years, with a range from 20–45 years (Table 1). The average net body weight gain during pregnancy was 14.0 kg. In total, 87.9% of the women were primiparous and the multiparous women had at most three children. More than 70% of the women had completed more than a high school education and had middle (50,000–100,000 Yuan) or high household (>100,000 Yuan) income levels. None of the women reported smoking or drinking alcohol during pregnancy, although 29.1% reported being exposed to passive smoking during pregnancy. Of the 282 infants included, 52% were boys. The mean gestational age was 39 weeks (range 35–43 weeks), and the mean birth weight was 3310 g (range 2210–4500 g).

All of the women had detectable urinary Cd concentrations in each trimester, with a median of 0.54 µg/g creatinine. Both the unadjusted (*p* < 0.001) and creatinine adjusted (*p* = 0.006) urinary Cd concentrations were significantly different across the three pregnancy trimesters. The unadjusted median urinary Cd concentrations decreased between the 1st and 2nd trimesters, while the median in the 2nd and 3rd trimester levels were similar. In contrast, the creatinine adjusted urinary Cd concentrations increased between the 1st and 2nd trimester of pregnancy and were similar in the 2nd and 3rd trimester (Table 2). The geometric mean of creatinine adjusted Cd levels in maternal urine was 0.51, 0.59, and 0.61 µg/g creatinine at the 1st, 2nd, and 3rd trimesters, respectively.

Creatinine adjusted urinary Cd levels were moderately to highly correlated at 1st and 2nd trimesters (*r* = 0.65), 2nd and 3rd trimesters (*r* = 0.55), and 1st and 3rd trimesters (*r* = 0.43) (all *p* < 0.05). There were no significant differences in each trimester between the creatinine adjusted Cd levels in maternal urine samples from the women who delivered male or female infants (see Supplementary Materials Table S1).

Table 3 shows the associations between birth size and the log-transformed creatinine adjusted Cd levels assessed using GEE models. Among all the newborns, we observed inverse associations between birth weight and maternal creatinine adjusted Cd concentrations at the 1st and 2nd trimester, although these associations were not significant. None of the maternal creatinine adjusted Cd levels in the three trimesters was associated with birth length. Both the 1st and 2nd trimester creatinine adjusted Cd levels were borderline significantly associated with ponderal index (β = −0.04; 95% CI: −0.07, 0.00 for 1st trimester, and β = −0.05; 95% CI: −0.10, 0.00 for 2nd trimester). When the exposure coefficients were estimated jointly, there was no significant difference in associations between maternal Cd levels across the three trimesters and birth weight, length, and ponderal index among all the infants (*p*_int_ = 0.34, 0.94, and 0.12 respectively).

We then conducted stratified analyses by sex (Table 3). In girls, we observed a significant inverse association between birth weight and creatinine adjusted Cd levels measured at the 1st trimester, and the estimated association was −116.99 g (95% CI: −208.87, −25.11) birth weight per 1-log_e_-unit increase in creatinine adjusted Cd levels. Creatinine adjusted Cd levels at the 2nd trimester were also negatively associated with birth weight in girls and the magnitude of this effect was similar to that of the 1st trimester (β = −115.78; 95% CI: −246.13, 14.56), although not statistically significant. There was no significant association between the 3rd trimester creatinine adjusted Cd levels and birth weight in girls. No significant associations were found between birth length in girls and maternal Cd levels measured during any of the trimesters. Results in girls for associations of ponderal index with creatinine adjusted Cd levels measured during the 1st (β = −0.05; 95% CI: −0.10, 0.00) and the 2nd (β = −0.07; 95% CI: −0.13, 0.00) trimester showed similar patterns to those observed for birth weight, with no association apparent for exposures measured at the 3rd trimester (Table 3). Tests of varying exposure effect showed the exposure coefficients for ponderal index were significantly different across three trimesters (*p*_int_ = 0.02), and borderline significantly different for birth weight (*p*_int_ = 0.08).

In boys, there was no evidence of an association between creatinine adjusted maternal urinary Cd levels and any birth size parameter, including for levels measured at any trimester (Table 3).

In sensitivity analyses, we first excluded five women whose gestational age was less than 37 weeks, but it did not markedly change any of the effect estimates (change < 5%) for creatinine adjusted maternal Cd on birth size (see Supplementary Materials Table S2). We then added calcium supplementation factor to the models, and the overall patterns of associations between maternal Cd levels and birth size were essentially unchanged (see Supplementary Materials Table S3). We also ran GEE models that incorporated log-transformed creatinine concentrations as a covariate, and the adjustment had little influence on associations between maternal Cd and birth size, although the birth weight estimate for 3rd trimester urinary Cd levels decreased somewhat (see Supplementary Materials Table S4).

## 4. Discussion

In this cohort study, we evaluated associations between birth size and Cd exposure measured at three trimesters of pregnancy. We found that maternal urine Cd levels varied moderately over pregnancy, it increased between the 1st and 2nd trimester of pregnancy and were similar in the 2nd and 3rd trimester. There were no apparent associations for boys alone, but among girls, we observed inverse associations between first trimester creatinine adjusted urinary Cd levels and birth weight and ponderal index.

Blood Cd levels and urinary cadmium are the most commonly used biomarkers of exposure. It is generally considered that blood Cd levels are indicative of recent exposure rather than whole-body burdens, and urine Cd levels may reflect 6–38 years status of exposure. However, our findings showed that the levels of urinary Cd were variable throughout the three trimesters.

Many studies have assessed the effects of maternal Cd exposure, but typically have relied on only one biosample measurement collected at varying times during pregnancy, at delivery, or on measurements collected from cord blood or placenta. There are few prior studies that have measured Cd exposure more than once during gestation. A recent study [14] of 1835 pregnant women from across Canada showed that there was no association between the average of the 1st and 3rd trimester measures of maternal blood Cd and SGA at birth. However, we are not aware of previous studies that have reported associations between serial maternal urinary Cd levels and birth outcomes. In a cohort study in rural Bangladesh [13] that measured maternal Cd exposure using one spot urine sample collected at the first trimester of pregnancy (Gestation Week 8), an inverse association was observed between maternal Cd exposures and birth weight, head circumference, and chest circumference, which is similar to our study was only observed in girls. Our findings of an association between maternal Cd exposure in the earlier stage of pregnancy and birth size among girls are broadly consistent with these results.

The fetus has different rates of growth at different stages of gestation, and environmental exposures occurring at different stages of pregnancy may have very different impacts on fetal development and birth outcomes. Although there have been few studies that have assessed longitudinal measurements of Cd over the course of pregnancy and birth outcomes, the plausibility that early pregnancy in particular may be a period of heightened susceptibility and a critical window has been noted for other environmental exposures, such as polycyclic aromatic hydrocarbons (PAHs). PAH exposure has been found to exert the greatest adverse effect on fetal growth during the first trimester, with inverse associations observed for the fetal growth ratio, birth weight, and birth length [20]. An animal study evaluating the effect of maternal undernutrition also provided direct evidence that fetal growth trajectory was determined in early pregnancy [21]. We observed a similar finding that Cd exposure in the third trimester had no effect on size at birth. The varying exposure effect tests also showed the overall difference among the three trimesters was driven by the effects observed in the first and second vs. third trimester. Thus, the effect of Cd exposure at earlier pregnancy stages was independent of the late stage Cd exposure. Combined, these data provide evidence that earlier pregnancy is the critical window of fetal susceptibility to adverse effects on birth size resulting from Cd exposure.

We found a sex difference in the associations between creatinine adjusted maternal urinary Cd levels and birth weight and ponderal index, which were only apparent in girls. This is consistent with previous research that has suggested sex differences in the disposition and patterns of toxicity for a variety of different metals [22]. Significant inverse associations between prenatal Cd exposure and birth anthropometry were found by Rollin [23] in female neonates but not in male neonates. The Omega Study [24] had reported that females had reduced birth length with greater U-Cd tertile, whereas males birth length marginally increased (β (95% CI) females: low = reference, middle = −0.59 cm (−1.37, 0.19), high = −0.83 cm (−1.69, 0.02), *p*-trend = 0.08; males: low = reference, middle = 0.18 cm (−0.59, 0.95), high = 0.78 cm (−0.04, 1.60), *p*-trend = 0.07; *p* for interaction = 0.03). Caroline M. Taylor [25] also found that maternal blood Cd level was adversely associated with birth weight, and the first and the second percentages of the mean-heel length in girls but not in boys in adjusted regression models. For Cd in particular, female-specific effects have been shown including increased absorption of Cd at low iron stores, estrogenic effects [22], and evidence of significantly higher Cd concentrations than male infants in the placenta [26]. The sex-specific effects of early life Cd exposure on DNA methylation may also be a possible mechanism by which maternal Cd exposure affects size at birth [26,27]. It has been shown that in girls maternal Cd exposure resulted in overrepresentation of methylation changes in genes associated with organ development, morphology and mineralization of bone, whereas changes in boys were found in cell death-related genes [27].

Previous study [28,29,30,31] suggested that urinary creatinine was more affected by age, gender, body size and meat intake. Since many studies did not focus on the changes of creatinine levels during each stage of pregnancy, we hypothesize that the changes of creatinine levels during each stage of pregnancy are also due to external factors such as body size, meat intake. The increase of urinary creatinine during pregnancy may also due to physiological loss of muscle caused by decreased activity during pregnancy. These external factors are almost reflected in all pregnant women. Our study population is pregnant women aged 20 years or older, and most of them are non-smokers. The changes of body size during pregnancy among them have the same trends. In this cohort study, creatinine adjustment is appropriate. Our findings further showed that the levels of urinary Cd were variable throughout the three trimesters. In our study population, the creatinine adjusted urinary Cd levels tended to increase during pregnancy. A study of 120 pregnant women in Spain [32] reported that there were no significant changes in creatinine adjusted urinary Cd concentrations at the 6th, 10th, 26th, and 30th weeks of pregnancy; however, it was suggested by the authors that increased dietary intake of calcium during the last trimester of pregnancy could have influenced these findings. The urinary Cd concentrations of the population in that study [32] ranged from 0.05 to 3.79 µg/g creatinine (geometric mean 0.49 µg/g creatinine), which is lower than but overlaps with exposure levels in our study population. Compared with other general populations, the pregnant women in our study had higher levels of urinary Cd than those in the USA (U.S. Centers for Disease Control and Prevention [33] (median, 0.22 µg/g creatinine; 75th–95th percentiles, 0.38–0.85 µg/g creatinine), and slightly higher levels than those reported in a study of 229 type 2 diabetic patients (mean, 0.38 µg/g creatinine; range, 0.05–4.17 µg/g creatinine) in Shanghai, China [34].

The main strengths of our study include the prospective study design and the assessment of maternal Cd levels in three urine samples collected from different trimesters, which enabled us to evaluate trimester-specific associations with birth size and evaluate critical windows of susceptibility during the pregnancy. In addition, we collected data on a broad array of potential confounders, including maternal BMI before pregnancy, net weight gain during pregnancy, and passive smoking. Our study also had some limitations. First, the sample size was modest, which was due to labor and cost limitations. A second limitation was that we did not adjust for dietary factors. However, we have controlled for calcium supplementation, and found it has almost no effect on the pattern of associations of maternal Cd concentrations and birth size.

## 5. Conclusions

We observed that maternal Cd exposure during the earlier stage of pregnancy was inversely associated with birth weight and ponderal index, which was apparent in girls but not boys. While these findings will need verification in other populations, our data suggest that cadmium exposure among young women and women of childbearing age should be as low as possible in order to keep the body burden as low as possible.

## Figures and Tables

**Table 1:** Characteristics of the study population of mother-infant pairs.

Characteristics	*n*	Mean ± SD (Range) or Percent
**Mothers**		
Age (years)	282	28.8 ± 3.4 (20–45)
Weight before pregnancy (kg)	282	54.1 ± 7.3 (40–82)
Height (cm)	282	161 ± 5 (150–172)
BMI before pregnancy (kg/m^2^)		20.8 ± 2.6 (15.6–29.7)
<18.5	53	18.8
18.5–23.9	193	68.4
≥24	36	12.8
Pregnancy net weight gain (kg)	282	14.0 ± 5.4 (0.8–50)
Parity	282	1.1 ± 0.3 (1–3)
primiparous	248	87.9
multiparous	34	12.1
Education		
Less than high school	19	6.7
High school	56	19.9
More than high school	207	73.4
Household income		
<50,000 Yuan per year	65	23.1
50,000–100,000 Yuan per year	121	42.9
>100,000 Yuan per year	96	34
Tobacco smoking (yes/no)	282	0/282
Passive smoking (yes/no)	282	82/200
Alcohol drinking (yes/no)	282	0/282
Calcium supplementation	282	254/28
**Infants**		
Sex		
Male	146	51.8
Female	136	48.2
Gestational age (weeks)	282	39.0 ± 1.2 (35–43)
Birth weight (g)	282	3321.6 ± 386.0 (2210–4500)
Birth length (cm)	282	50.1 ± 1.4 (45–54)

**Table 2:** Maternal Cd levels in urine samples collected during each pregnancy trimester from women in Wuhan, China.

Cd Concentrations	*n*	GM ^a^	AM ^b^	Percentile		
				25th	50th	75th
Unadjusted (µg/L)						
1st trimester	279	0.48	0.72	0.27	0.54	0.96
2nd trimester	246	0.35	0.50	0.20	0.36	0.65
3rd trimester	276	0.35	0.46	0.21	0.37	0.62
Creatinine adjusted (µg/g creatinine)						
1st trimester	279	0.51	0.62	0.36	0.49	0.77
2nd trimester	246	0.59	0.68	0.41	0.56	0.79
3rd trimester	276	0.61	0.74	0.39	0.57	0.92

^a^ geometric mean; ^b^ arithmetic mean.

**Table 3:** Generalized estimating equation models ^a^ evaluating trimester-specific associations between birth size and log-transformed creatinine adjusted maternal urinary Cd levels (µg/g creatinine).

Variable	*n*	Birth Weight (g)	*p*-Value	Birth Length (cm)	*p*-Value	Ponderal Index	*p*-Value
		β (95% CI) ^b^		β (95% CI) ^c^		β (95% CI) ^d^	
All ^a^							
1st trimester	238	−56.91 (−124.04, 10.22)	0.09	−0.01 (−0.25, 0.24)	0.94	−0.04 (−0.07, 0.00)	0.06
2nd trimester	238	−62.89 (−152.50, 26.77)	0.20	0.01 (−0.32, 0.34)	0.94	−0.05 (−0.10, 0.00)	0.05
3rd trimester	238	6.01 (−56.69, 68.71)	0.85	0.05 (−0.18, 0.28)	0.67	0.01 (−0.03, 0.04)	0.91
*p*_int_-Value ^e^			0.34		0.94		0.12
Girls							
1st trimester	113	−116.99 (−208.87, −25.11)	0.01	−0.21 (−0.56, 0.14)	0.24	−0.05 (−0.10, 0.00)	0.05
2nd trimester	113	−115.78 (−246.13, 14.56)	0.08	−0.14 (−0.64, 0.35)	0.57	−0.07 (−0.13, 0.00)	0.05
3rd trimester	113	47.82 (−37.72, 133.35)	0.27	0.08 (−0.24, 0.41)	0.61	0.03 (−0.01, 0.08)	0.09
*p*_int_-Value ^e^			0.08		0.56		0.02
Boys							
1st trimester	125	−1.23 (−100.37, 97.90)	0.98	0.21 (−0.14, 0.55)	0.24	−0.03 (−0.09, 0.03)	0.31
2nd trimester	125	−23.82 (−149.98, 102.34)	0.71	0.12 (−0.32, 0.56)	0.60	−0.04 (−0.12, 0.04)	0.33
3rd trimester	125	−41.65 (−133.82, 50.53)	0.38	−0.02 (−0.35, 0.30)	0.88	−0.02 (−0.08, 0.04)	0.45
*p*_int_-Value ^e^			0.71		0.50		0.93

^a^ Adjusted for maternal age, BMI before pregnancy, net weight gain during pregnancy, maternal education, passive smoking, and gestational age. Models for all the newborns were also adjusted for sex. Maternal height was used instead of BMI before pregnancy for birth length analyses; ^b^ Coefficients are expressed as birth weight change (g) per unit increase in log(µg/g creatinine) Cd; ^c^ Coefficients are expressed as birth length change (cm) per unit increase in log(µg/g creatinine) Cd; ^d^ Coefficients are expressed as ponderal index change (kg/m^3^) per unit increase in log(µg/g creatinine) Cd; ^e^ Score test of homogeneity of coefficients.

## Supplementary Materials

The following are available online at www.mdpi.com/1660-4601/14/1/58/s1, Table S1: Cd levels in urinary samples of women who delivered boys or girls, Table S2: Multivariable linear regression analyses of the association between maternal urinary cadmium and newborn anthropometrics with creatinine as a covariate in the model, Table S3: Generalized estimating equation models including adjustment for calcium supplementation in the evaluation of trimester-specific associations between birth size and log-transformed creatinine adjusted maternal urinary Cd levels (µg/g creatinine), Table S4: Generalized estimating equation models evaluating trimester-specific associations between birth size and log-transformed creatinine adjusted maternal urinary Cd levels (µg/g creatinine) with creatinine as a covariate.
