# Supplementary material for: Critical Windows of Prenatal Exposure to Cadmium and Size at Birth
Source: Int J Environ Res Public Health. 2017 Jan 9;14(1):58. doi: 10.3390/ijerph14010058 (PMC5295309; doi:10.3390/ijerph14010058)
Supplement: Supplementary file 1 [file ijerph-14-00058-s001.pdf]

Supplementary Materials: Critical Windows of Prenatal Exposure to Cadmium and Size at Birth

Lu Cheng, Bin Zhang, Tongzhang Zheng, Jie Hu, Aifen Zhou, Bryan A. Bassig, Wei Xia, David A. Savitz, Stephen Buka, Chao Xiong, Joseph M. Braun, Yaqi Zhang, Yanqiu Zhou, Xinyun Pan, Chuansha Wu, Youjie Wang, Zhengmin Qian, Aimin Yang, Megan E. Romano, Kunchong Shi, Shunqing Xu, Yuanyuan Li

Table S1. Cd levels in urinary samples of women who delivered boys or girls.

Cd Concentrations	Girls		Boys		*p*-Value [a]
	n	Median	*n*	Median	
Unadjusted (μg/L)					
1st trimester	135	0.56	144	0.50	0.73
2nd trimester	115	0.36	131	0.34	0.82
3rd trimester	135	0.39	141	0.35	0.27
Creatinine adjusted (μg/g creatinine)					
1st trimester	135	0.53	144	0.47	0.08
2nd trimester	115	0.59	131	0.55	0.42
3rd trimester	135	0.64	141	0.54	0.15

[a] wilcoxon rank sum test.

Table S2. Multivariable linear regression analyses of the association between maternal urinary cadmium and newborn anthropometrics with creatinine as a covariate in the model.

Variable	*n*	Birth Weight (g) β (95% CI) [b]	*p*-Value	Birth Length (cm) β (95% CI) [c]	*p*-Value	Ponderal Index β (95% CI) [d]	*p*-Value
All [a]							
1st trimester	238	−56.29 (−124.15, 11.58)	0.10	0.01 (−0.24, 0.26)	0.94	−0.04 (−0.08, 0.00)	0.06
2nd trimester	238	−62.21 (−152.49, 28.06)	0.18	0.02 (−0.31, 0.35)	0.91	−0.05 (−0.10, 0.00)	0.05
3rd trimester	238	6.05 (−57.27, 69.37)	0.85	0.05 (−0.18, 0.28)	0.65	0.01 (−0.03, 0.04)	0.77
p_{int}-Value [e]			0.35		0.98		0.12
Girls							
1st trimester	113	−117.08 (−210.71, −24.89)	0.01	−0.20 (−0.56, 0.15)	0.26	−0.05 (−0.10, 0.00)	0.05
2nd trimester	113	−117.20 (−248.79, 14.38)	0.08	−0.14 (−0.64, 0.35)	0.58	−0.07 (−0.14, 0.00)	0.06
3rd trimester	113	48.27 (−38.26, 134.80)	0.27	0.09 (−0.23, 0.42)	0.56	0.03 (−0.01, 0.08)	0.15
p_{int}-Value [e]			0.10		0.56		0.02
Boys							
1st trimester	125	−4.78 (−105.07, 95.52)	0.93	0.22 (−0.13, 0.57)	0.22	−0.04 (−0.10, 0.03)	0.25
2nd trimester	125	−27.73 (−154.94, 99.49)	0.67	0.12 (−0.33, 0.57)	0.60	−0.04 (−0.12, 0.04)	0.29
3rd trimester	125	−42.26 (−135.28, 50.83)	0.37	−0.03 (−0.36, 0.29)	0.85	−0.02 (−0.08, 0.04)	0.47
p_{int}-Value [e]			0.73		0.44		0.89

[a] Adjusted for maternal age, BMI before pregnancy, net weight gain during pregnancy, maternal education, passive smoking, gestational age. Models for all the newborns were also adjusted for sex. Maternal height was used instead of BMI before pregnancy for birth length analyses; [b] Coefficients are expressed as birth weight change (g) per unit increase in log(μg/g creatinine) Cd; [c] Coefficients are expressed as birth length change (cm) per unit increase in log(μg/g creatinine) Cd; [d] Coefficients are expressed as ponderal index change (kg/m^3) per unit increase in log(μg/g creatinine) Cd; [e] Score test of homogeneity of coefficients.

Table S3. Generalized estimating equation models [a] including adjustment for calcium supplementation in the evaluation of trimester-specific associations between birth size and log-transformed creatinine adjusted maternal urinary Cd levels (μg/g creatinine).

Variable	*n*	β (95% CI) [b]	*p*-Value	β (95% CI) [c]	*p*-Value	β (95% CI) [d]	*p*-Value
All [a]							
1st trimester	238	−57.09 (−124.27, 10.07)	0.10	0.01 (−0.25, 0.23)	0.94	−0.04 (−0.07, 0.00)	0.07
2nd trimester	238	−62.46 (−152.16, 27.23)	0.17	0.01 (−0.32, 0.33)	0.94	−0.05 (−0.10, 0.00)	0.05
3rd trimester	238	6.47 (−56.30, 69.23)	0.84	0.05 (−0.18, 0.28)	0.67	0.01 (−0.03, 0.04)	0.74
p_{int}-Value [e]			0.34		0.94		0.12
Girls							
1st trimester	113	−116.96 (−208.97, −24.96)	0.01	−0.20 (−0.56, 0.14)	0.25	−0.05 (−0.10, 0.00)	0.05
2nd trimester	113	−115.87 (−246.39, 14.66)	0.08	−0.15 (−0.64, 0.35)	0.56	−0.07 (−0.13, 0.00)	0.06
3rd trimester	113	47.71 (−37.95, 133.37)	0.28	0.08 (−0.24, 0.41)	0.62	0.03 (−0.01, 0.08)	0.16
p_{int}-Value [e]			0.10		0.57		0.02
Boys							
1st trimester	125	−1.74 (−101.06, 97.59)	0.97	0.22 (−0.13, 0.56)	0.22	−0.03 (−0.09, 0.03)	0.29
2nd trimester	125	−223.22 (−149.61, 103.16)	0.72	0.11 (−0.33, 0.56)	0.63	−0.04 (−0.11, 0.04)	0.36
3rd trimester	125	−40.85 (−133.33, 51.63)	0.39	−0.04 (−0.36, 0.28)	0.81	−0.02 (−0.08, 0.04)	0.50
p_{int}-Value [e]			0.71		0.40		0.91

[a] Adjusted for maternal age, BMI before pregnancy, net weight gain during pregnancy, maternal education, passive smoking, gestational age, and calcium supplementation. Models for all the newborns were also adjusted for sex. Maternal height was used instead of BMI before pregnancy for birth length analyses; [b] Coefficients are expressed as birth weight change (g) per unit increase in log(μg/g creatinine) Cd; [c] Coefficients are expressed as birth length change (cm) per unit increase in log(μg/g creatinine) Cd; [d] Coefficients are expressed as ponderal index change (kg/m^3) per unit increase in log(μg/g creatinine) Cd; [e] Score test of homogeneity of coefficients.

Table S4. Generalized estimating equation models [a] evaluating trimester-specific associations between birth size and log-transformed creatinine adjusted maternal urinary Cd levels (μg/g creatinine) with creatinine as a covariate.

Variable	*n*	Birth Weight (g) β (95% CI) [b]	*p*-Value	Birth Length (cm); β (95% CI) [c]	*p*-Value	Ponderal Index; β (95% CI) [d]	*p*-Value
All [a]							
1st trimester	238	−56.12 (−123.39, 11.14)	0.10	−0.01 (−0.25, 0.24)	0.94	−0.04 (−0.07, 0.00)	0.07
2nd trimester	238	−62.54 (−152.03, 26.95)	0.17	0.01 (−0.32, 0.34)	0.94	−0.05 (−0.10, 0.00)	0.05
3rd trimester	238	−16.95 (−83.02, 49.11)	0.62	−0.07 (−0.31, 0.18)	0.58	0.01 (−0.03, 0.04)	0.81
p_{int}-Value [e]			0.62		0.89		0.13
Girls							
1st trimester	113	−115.32 (−206.54, −24.10)	0.01	−0.21 (−0.56, 0.14)	0.24	−0.05 (−0.09, 0.00)	0.05
2nd trimester	113	−120.63 (−250.30, 9.05)	0.07	−0.15 (−0.65, 0.34)	0.57	−0.07 (−0.14, 0.00)	0.05
3rd trimester	113	16.23 (−71.90, 104.36)	0.72	−0.03 (−0.37, 0.30)	0.61	0.03 (−0.02, 0.07)	0.25
p_{int}-Value [e]			0.14		0.80		0.02
Boys							
1st trimester	125	−3.23 (−103.40, 96.94)	0.95	0.22 (−0.13, 0.57)	0.22	−0.04 (−0.10, 0.03)	0.26
2nd trimester	125	−21.84 (−148.90, 105.20)	0.74	0.11 (−0.33, 0.55)	0.63	−0.04 (−0.11, 0.04)	0.38
3rd trimester	125	−52.46 (−152.74, 47.81)	0.31	−0.16 (−0.51, 0.19)	0.88	−0.01 (−0.08, 0.05)	0.64
p_{int}-Value [e]			0.61		0.16		0.83

[a] Adjusted for maternal age, BMI before pregnancy, net weight gain during pregnancy, maternal education, passive smoking, gestational age, and log-transformed creatinine concentrations. Models for all the newborns were also adjusted for sex. Maternal height was used instead of BMI before pregnancy for birth length analysis; [b] Coefficients are expressed as birth weight change (g) per unit increase in log(μg/g creatinine) Cd; [c] Coefficients are expressed as birth length change (cm) per unit increase in log(μg/g creatinine) Cd; [d] Coefficients are expressed as ponderal index change (kg/m^3) per unit increase in log(μg/g creatinine) Cd; [e] Score test of homogeneity of coefficients.

© 2017 by the authors. Submitted for possible open access publication under the terms and conditions of the Creative Commons Attribution (CC-BY) license (http://creativecommons.org/licenses/by/4.0/).
